# Validation and factor analysis of mother-infant bonding questionnaire in pregnant and postpartum women in Japan

**DOI:** 10.1186/s12888-016-0933-3

**Published:** 2016-07-07

**Authors:** Masako Ohara, Takashi Okada, Chika Kubota, Yukako Nakamura, Tomoko Shiino, Branko Aleksic, Mako Morikawa, Aya Yamauchi, Yota Uno, Satomi Murase, Setsuko Goto, Atsuko Kanai, Tomoko Masuda, Norio Ozaki

**Affiliations:** Department of Psychiatry, Nagoya University Graduate School of Medicine, 65 Tsurumai-cho, Showa-ku, Nagoya, Aichi 466-8550 Japan; Sugiyama Jogakuen University, Nagoya, Japan; Graduate School of Education and Human Development, Nagoya University, Nagoya, Japan; Graduate School of Law, Nagoya University, Nagoya, Japan

## Abstract

**Background:**

The Mother-Infant Bonding Questionnaire (MIBQ) has been widely used to assess maternal emotional involvement with infants. Although the reliability and validity of the MIBQ in the postpartum period has been confirmed, it remains unclear whether the MIBQ is appropriate to assess maternal bonding in both pregnancy and the postpartum period over time. Our study were aimed to 1) examine the reliability and validity of the MIBQ for clinical use among pregnant and postpartum women; and 2) examine the factor structure of the items, create subscales, and confirm the stability of the MIBQ in the pregnancy and postpartum periods.

**Methods:**

Participants (*n* = 751, mean age 32.1 ± 4.4 years) completed the MIBQ and the Edinburgh Postnatal Depression Scale (EPDS) in early pregnancy (before week 25), in late pregnancy (around week 36), 5 days after delivery, and 1 month after delivery. We randomly divided participants into two sample sets. We conducted an exploratory factor analysis of the nine MIBQ items using data from one group of mothers (Group 1; *n* = 376) in all four periods. The factor structure derived from the exploratory factor analysis was confirmed by a confirmatory factor analysis in the second group (Group 2; *n* = 375) of mothers in all four periods.

**Results:**

Exploratory factor analysis yielded two factors: Lack of Affection (LA) and Anger and Rejection (AR). Confirmatory factor analysis demonstrated that LA and AR factors existed for the MIBQ in all periods. Cronbach’s alpha coefficients were 0.879 and 0.584, respectively. The scores for LA and AR were significantly correlated over the four time periods. Mothers with higher AR scores on the MIBQ at any of the four periods had higher scores on the EPDS.

**Conclusions:**

The MIBQ has two subscales regardless of the timing of the assessment. The MIBQ is appropriate for pregnant as well as postpartum women to assess maternal bonding toward the fetus and infant.

## Background

Maternal emotional feelings and loving attitude toward their infant lead to establishment of an effective attachment system. This important psychological process was named “bonding” [1]. Kumar reported that some mothers showed a delay in the development of maternal bonding, resulting in infant neglect or impulses to harm the infant [2]. This finding was supported by subsequent observational studies. For example, cross-sectional studies suggested that poor mother-infant bonding was associated with less interactive behaviors [3, 4]. Kitamura et al. performed path analysis to elucidate causal relationships between maternal bonding and abusive parenting, and indicated that bonding failure in the postpartum period predicted abusive parenting [5]. In addition, a longitudinal study also showed that bonding impairment in the postpartum period was related to insecure parenting behaviors [6].

Maternal bonding starts to develop before a child is born [7]. Observational studies examined whether negative maternal involvement with the fetus was correlated with adverse effects on the infant’s outcome. Alhusen et al. reported that the children whose mothers reported lower maternal emotional involvement during pregnancy had less optimal early childhood development and less secure interactions with their mothers [8].

In addition, it has been suggested that maternal bonding in pregnancy predicts maternal bonding in the postpartum period. In a longitudinal study, women with more feelings of closeness and tenderness toward their fetuses in pregnancy reported more feelings of pleasure and affiliation in the relationship with their infants [9, 10]. These results suggest that maternal bonding disturbances start in pregnancy and are related to impairments in the mother-child relationship, which may result in maltreatment or child abuse. To ensure a positive relationship between the mother and infant in the postpartum period, it is essential to detect maternal bonding disturbances earlier in the perinatal period, and to follow up on the status of these disturbances. Therefore, it is important to assess maternal bonding during pregnancy and the postpartum period over time, using the same instruments. However, there have been no appropriate instruments to assess maternal bonding in both pregnancy and the postpartum period over time. The Postpartum Bonding Questionnaire (PBQ) has been standardized for the postpartum period [11]. Some items on the PBQ are applicable only to postpartum women (e.g., “I feel happy when my baby smiles or laughs). The Maternal Antenatal Attachment Scale (MAAS) is a widely used measure of maternal-fetal attachment during pregnancy [12]. Some items on the MAAS are applicable only to pregnant women (e.g., “Frequent/infrequent picturing of the fetus in my imagination”).

Kumar developed a self-report screening scale, based on the mother’s narrative accounts [2]. This scale was named the Mother-Infant Bonding Questionnaire (MIBQ), which has nine items: “loving”, “disappointed”, “neutral or felt nothing”, “possessive”, “resentful”, “dislike”, “protective”, “joyful”, and “aggressive” [13, 14]. All items on the MIBQ are applicable during for the pregnancy and postpartum periods. The MIBQ does not require a high reading level, and takes only a few minutes to complete. Although the reliability and validity of the MIBQ in the postpartum period has been confirmed [13], it remains unclear whether the MIBQ is also appropriate during pregnancy.

Therefore, the aims of our study were as follows: 1) to examine the reliability and validity of the MIBQ for clinical use among pregnant and postpartum women; and 2) to examine the factor structure of the items, create subscales, and confirm the stability of the MIBQ in the pregnancy and postpartum periods.

## Methods

### Participants

Participants in this study were recruited from perinatal classes for pregnant women (starting before week 25 of pregnancy) at two obstetrical hospitals and one university hospital in central Nagoya, Japan (with a population of approximately 2 million) between August 2004 and March 2015. Mothers with current or past histories of mental illness were excluded from the study, as well as mothers with children born before week 32 of gestation. In addition, participants were required to be at least 20 years old and capable of understanding the Japanese language.

### Procedures

Pregnant women attending perinatal classes were given detailed information about the study design and methods. This information was given orally and on paper in the three hospitals. Women who agreed to cooperate in the study were asked to complete self-reporting questionnaires, which included social demographic questions, the MIBQ, and the Edinburgh Postnatal Depression Scale (EPDS) in early pregnancy before week 25 (T1), and return them by mail. After receiving the completed consent forms and questionnaires, the MIBQ and the EPDS questionnaires were sent again around week 36 of pregnancy (T2) and returned by mail. At day 5 and 1 month after delivery (T3, T4), the MIBQ and the EPDS were sent and returned by mail. A total of 990 women agreed to participate at the perinatal classes starting before week 25 of pregnancy; 751 (75.9 %) mothers completed all questionnaires for all periods.

### Measures

#### Mother-Infant Bonding Questionnaire (MIBQ)

The MIBQ is a self-reporting scale designed to assess maternal bonding with their baby during the postpartum period; it is composed of nine items: “loving”, “disappointed”, “neutral or felt nothing”, “possessive”, “resentful”, “dislike”, “protective”, “joyful”, and “aggressive” [13, 14]. The MIBQ is rated on a four-point Likert scale (from 0, “very much” to 3, “not at all”), with the scale of some items reversed. Total scores range from 0 to 27. A high score indicates worse mother to infant bonding. The reliability and validity of the MIBQ in the postpartum period have been reported [13]. Yamashita translated the MIBQ into Japanese, and then the Japanese version was retranslated back into English by a native English translator who was unaware of the original wording to confirm that the translation was consistent with the original meaning. This version was used in a previous clinical study in Japan [15]. The reliability and validity of the Japanese version of the MIBQ have not been reported.

#### Edinburgh Postnatal Depression Scale (EPDS)

The EPDS is a self-reporting questionnaire designed to assess postpartum depression; it is composed of 10 items scored on a four-point Likert scale [16]. Numerous studies have used this instrument during pregnancy and the postpartum period. The EPDS Japanese version showed good internal consistency (Cronbach’s alpha = 0.78) and test-retest reliability (Spearman’s correlation = 0.92) [17]. A score ≥9 was designated to screen for minor and major depressive episodes, with a sensitivity of 75 % and 82 % and a specificity of 93 % and 95 %, respectively [17, 18].

### Statistical analysis

First, participants (*n* = 751, mean age 32.1 ± 4.4 years) were randomly divided into two groups (Group 1, *n* = 376; Group 2, *n* = 375) using a random sampling technique. We calculated descriptive statistics for the nine items of the MIBQ as shown in Table 1. Most of the items were positively skewed (skew values greater than 1) at all time points. We therefore log-transformed all MIBQ scores for the subsequent factor analysis. We conducted an exploratory factor analysis (EFA) of nine items of the MIBQ using data from Group 1. Because all factors were considered dependent upon each other, the factor solution was sought after Promax rotation, which is an oblique rotation. The number of factors was determined by scree plot [19]. To create a subscale of the MIBQ, we extracted items for each subscale if they were loaded ≥0.3 on a particular factor but <0.3 on the other factors.

**Table 1:** Means and SDs of the MIBQ items at the four time points in Group 1

MIBQ items	T1		T2		T3		T4	
	M (SD)	Skewness	M (SD)	Skewness	M (SD)	Skewness	M (SD)	Skewness
1: loving	0.45 (0.65)	1.28	0.39 (0.59)	1.29	0.10 (0.35)	3.99	0.09 (0.30)	3.58
2: disappointed	0.06 (0.30)	5.87	0.05 (0.31)	6.45	0.07 (0.35)	5.61	0.09 (0.40)	5.47
3: neutral and felt nothing	0.42 (0.80)	1.75	0.44 (0.83)	1.82	0.26 (0.72)	2.89	0.19 (0.61)	3.36
4: possessive	1.09 (1.03)	0.45	0.90 (0.96)	0.69	0.59 (0.90)	1.32	0.62 (0.88)	1.26
5: resentful	0.04 (0.25)	7.08	0.04 (0.24)	6.18	0.07 (0.32)	5.07	0.24 (0.55)	2.22
6: dislike	0.10 (0.41)	4.71	0.11 (0.48)	4.70	0.07 (0.36)	5.68	0.13 (0.45)	3.79
7: protective	0.30 (0.60)	2.04	0.34 (0.61)	1.77	0.13 (0.42)	3.64	0.09 (0.32)	3.96
8: joyful	0.61 (0.82)	1.16	0.64 (0.81)	1.01	0.22 (0.54)	2.73	0.25 (0.52)	2.01
9: aggressive	0.06 (0.32)	5.57	0.04 (0.25)	8.10	0.03 (0.22)	9.49	0.09 (0.34)	4.29
Total	3.11 (3.03)	1.24	2.95 (2.93)	1.19	1.54 (2.30)	3.03	1.78 (2.45)	2.17

Each item is scored on a four-point Likert scale ranging from 0 to 3. Total scores can range from 0 to 27

*MIBQ* Mother-Infant Bonding Questionnaire, *M* mean, *SD* standard deviation, *T1* early pregnancy before week 25, *T2* late pregnancy around week 36, *T3* 5 days after delivery, *T4* 1 month after delivery

Second, the factor structure derived from the EFA was confirmed by a confirmatory factor analysis (CFA) in Group 2. The fit of each model with the data was examined in terms of chi-squared (CMIN), degree of freedom (df), comparative fit index (CFI), and root mean square error of approximation (RMSEA).

According to conventional criteria, CMIN/*df* < 2, CFI > 0.97, and RMSEA < 0.05 indicate a good fit, while CMIN/*df* < 3, CFI > 0.95, and RMSEA < 0.08 indicate an acceptable fit [20]. The Akaike Information Criterion (AIC) was used to compare different models; a model with an AIC score at least two points lower is regarded as a better model.

Cronbach’s alpha for the two hypothesized subscales was calculated to examine the internal reliability of the MIBQ.

Previous studies reported that maternal depressive mood associated with bonding disturbances [21–23]. In addition, in previous studies, EPDS was used as a way to assess construct validity for other instruments to assess bonding disturbances [14]. In accordance with previous research, we constructed a correlation matrix with the EPDS. This analysis provided evidence of construct validity.

All statistical analyses were conducted using the SPSS version 22.0 and Amos 21.0 (IBM Japan, Tokyo, Japan).

## Results

### Descriptive statistics

The means and SDs of all MIBQ items in early pregnancy before week 25 (T1), late pregnancy around week 36 (T2), 5 days after delivery (T3), and 1 month after delivery (T4) were relatively low (Table 1). In Table 1, total scores at T3 (1.54 ± 2.30) were lower than other points; T1 (3.11 ± 3.03), T2 (2.95 ± 2.93), and T4 (1.78 ± 2.45). Total scores decreased from T1 to T2. Total scores increased from T3 to T4. This is in line with previous report [24]. Most of the items were positively skewed. We therefore log-transformed all scores for MIBQ items before entering them into the EFA.

### Factor analysis of MIBQ items

All the log-transformed items of the MIBQ were entered into an EFA. This suggested a two-factor structure (Table 2). The first factor was loaded by five items: “loving”, “neutral or felt nothing”, “possessive”, “protective”, and “joyful”. These items reflected lack of positive affection and intimacy toward the baby. We named this factor Lack of Affection (LA). The second factor was loaded by two items: “resentful” and “dislike”. These items reflected a mother’s anger and rejection toward the baby. We named this factor Anger and Rejection (AR).

**Table 2:** Factor structure of the MIBQ items at early pregnancy before week 25 in Group 1

MIBQ items	Factor 1	Factor 2
8: joyful	**.819**	-.005
1: loving	**.810**	-.030
7: protective	**.718**	.104
4: possessive	**.517**	-.167
3: neutral or felt nothing	**.300**	.180
6: dislike	-.068	**.688**
5: resentful	-.010	**.682**
2: disappointed	.181	.299
9: aggressive	-.038	.253

Factor loadings ≥ 0.3 are bold

*MIBQ* Mother-Infant Bonding Questionnaire

**Fig. 1:**
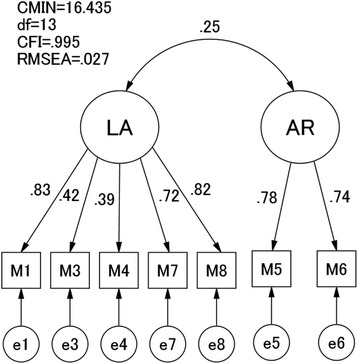
CFA of the MIBQ items at early pregnancy before week 25 (T1) in Group 2. CFA, confirmatory factor analysis; MIBQ, Mother-Infant Bonding Questionnaire; LA, Lack of Affection; AR, Anger and Rejection; CMIN, chi-squared; df, degree of freedom; CFI, comparative fit index; RMSEA, root mean square error of approximation; M, log-transformed score on each item of the MIBQ; e, error variable

The factor structure extracted in the EFA of the MIBQ was then subjected to a CFA using Group 2. The model derived from the EFA showed good fit with the data (CMIN/*df* = 1.264, CFI = 0.995, RMSEA = 0.027) (Fig. 1). The two latent factors were moderately correlated with each other.

To examine stability of the factor structure of the MIBQ, we conducted a series of CFAs using all the periods from Group 2. The path values and accompanying fit indices are shown in Table 3. The correlation between the LA and AR factors was moderate in all four periods. For all four periods, CMIN/*df* values were <2, indicating a good model of fit [20]. Examination of CFI and RMSEA revealed the current model to meet good fit criteria for all four periods. The fitness of the model to data was good for data from all four periods.

**Table 3:** CFA of the MIBQ items over the four time points in Group 2

MIBQ items	T1	T2	T3	T4
Lack of Affection (LA)				
1: Loving	0.83	0.85	0.71	0.65
3: Neutral or felt nothing	0.42	0.36	0.18	0.14
4: Possessive	0.39	0.45	0.37	0.37
7: Protective	0.72	0.72	0.7	0.52
8: Joyful	0.82	0.75	0.68	0.76
Anger and Rejection (AR)				
5: Resentful	0.78	0.75	0.3	0.57
6: Dislike	0.74	0.59	0.16	0.69
Covariance between LA and AR	0.25	0.34	0.23	0.29
CMIN/df	1.264	1.423	0.94	1.09
CFI	0.995	0.991	1	0.996
RMSEA	0.027	0.034	0	0.016
AIC	46.435	48.499	42.215	44.175

*CFA* confirmatory factor analysis, *MIBQ* Mother-Infant Bonding Questionnaire, *T1* early pregnancy before week 25, *T2* late pregnancy around week 36, *T3* 5 days after delivery, *T4* 1 month after delivery, *LA* lack of affection, *AR* Anger and Rejection, *CMIN*/*df* chi-squared/degree of freedom, *CFI* comparative fit index, *RMSEA* root mean square error of approximation

### Internal consistency of mother-infant bonding questionnaire

As for the combined data of Group 1, internal consistency (Cronbach’s alpha) of the five items belonging to LA was 0.879, whereas that of two items belonging to AR was 0.584, showing reasonable internal consistency.

### Construct validity of mother-infant bonding questionnaire

Both LA and AR subscales were positively correlated with the EPDS scores in all four periods (Table 4). Thus, negative affect of mothers correlated with worse bonding. The strongest correlations were between AR and EPDS, especially at 1 month postpartum.

**Table 4:** Pearson’s correlations of MIBQ subscale scores and EPDS, *n* = 751

		T1		T2		T3		T4		EPDS	EPDS	EPDS	EPDS
		LA	AR	LA	AR	LA	AR	LA	AR	T1	T2	T3	T4
T1	LA		0.21^**^	0.73^**^	0.20^**^	0.43^**^	0.06	0.48^**^	0.24^**^	0.14^**^			
	AR			0.20^**^	0.48^**^	0.07^*^	0.27^**^	0.07	0.28^**^	0.25^**^			
T2	LA				0.21^**^	0.46^**^	0.06	0.50^**^	0.22^**^		0.18^**^		
	AR					0.14^**^	0.36^**^	0.18^**^	0.43^**^		0.25^**^		
T3	LA						0.18^**^	0.66^**^	0.24^**^			0.17^**^	
	AR							0.14^**^	0.38^**^			0.25^**^	
T4	LA								0.26^**^				0.23^**^
	AR												0.33^**^

*MIBQ*, Mother-Infant Bonding Questionnaire, *EPDS* Edinburgh Postnatal Depression Scale, *T1* early pregnancy before week 25, *T2* late pregnancy around week 36, *T3* 5 days after delivery, *T4* 1 month after delivery, *LA* lack of affection, *AR* Anger and Rejection

*p < 0.05

**p < 0.01

### Test-retest reliability of mother-infant bonding questionnaire

Pearson’s correlations of MIBQ subscales are shown in Table 4. The scores of LA were significantly correlated over the four time points (all *p* < 0.01). Those of AR were also significantly correlated over the four time points (all *p* < 0.01).

## Discussion

To the best of our knowledge, the present study is the first to validate the MIBQ, examine the factor structure of the items, create subscales, and confirm stability in both the pregnancy and postpartum periods.

The reliability and validity of the MIBQ were examined among subjects during pregnancy and in the postpartum period. In this cohort, the two-factor structure, LA and AR, was confirmed in all four periods. The internal consistencies of the two factors were reasonable. The two subscales of the MIBQ were positively correlated with EPDS scores over time and demonstrated acceptable construct validity. In addition, the scores of LA were significantly correlated over the four time points, as were the scores of AR. These results demonstrated the stability of the MIBQ over the periods of the current study. Therefore, the MIBQ should be considered a reasonable scale to assess maternal emotional involvement with the fetus and infant during pregnancy and in the postpartum period.

The strength of our study is that we conducted an EFA for half of the participants (Group 1; *n* = 376) and confirmed stability of factor structure in the other half (Group 2; *n* = 375) to compare our four time points. The present study suggested that the MIBQ items fit the two-factor model for data in EFA. Taylor et al. also investigated the psychometric properties of the MIBQ [13]. He examined inter-item correlations of the MIBQ by using principal components analysis, and proposed that the original nine-item Kumar’s MIBQ score fit a one-factor model after discarding one item (item 4; possessive). We also tested Taylor’s one-factor model. The results did not show goodness-of-fit with our data (CMIN/*df* = 13.42, CFI = 0.691, RMSEA = 0.182, AIC = 300.328). Therefore, our two-factor model had an adequate fit to our data and was superior in statistical significance to the one-factor model.

According to our two-factor model, the MIBQ is likely to detect LA and AR. These subscales correspond with the factors that were proposed in previous studies using other instruments that assessed maternal bonding (PBQ [11], Mother-to-Infant Bonding Scale [5, 14]). Our two subscales correspond with “Impaired bonding” and “Rejection and Anger” in the 25-item PBQ by Brockington et al. that revealed a four-factor structure. Brockington et al. examined PBQ and proposed the following four-factor model: Scale 1 is “Impaired bonding”; Scale 2 is “Rejection and Anger”; Scale 3 may be useful in anxious mothers; and Scale 4 signaled the presence of incipient abuse [11]. In addition, our two subscale correspond with “Lack of Affection” and “Anger and Rejection” in the 10-item Japanese version of the Mother-to-Infant Bonding Scale by Yoshida et al. and Kitamura et al. that revealed a two-factor structure [5, 14].

It is should be emphasized that the EPDS showed correlations with both LA and AR. This means that both LA and AR were associated with the psychopathology of depressive symptoms. Furthermore, the correlation was especially large in the AR factor over the four periods. This finding suggests that depressive symptoms might be especially linked with anger and rejection toward a child. This study elucidated the associations between depressive symptoms and bonding disturbances. This is in line with previous reports. For example, Moehler et al. examined the relationship between postnatal depression and maternal bonding to the infant with the PBQ and EPDS using multivariate analysis. They reported that maternal depressive symptoms in the postnatal period were associated with long-term impairment of mother-child bonding [25]. In addition, Sockol also investigated correlates of mother-infant bonding among postpartum women with the PBQ and EPDS using multiple regression analysis. They reported that one of the strongest predictors of impairments in mother-infant bonding was maternal depressive symptom level [26]. However, there are numerous variables known to be associated with bonding disturbances and depressive symptoms. Further study is needed to elucidate these two issues.

Several limitations of this study need to be addressed. First, participants of this study were recruited from perinatal classes for pregnant women, and they participated in this study voluntarily. Therefore, this sample may not be representative of the total population. Second, this study was based only on self-administered questionnaires without diagnostic procedures based on external criteria. Therefore, criterion validity was not investigated in this study.

## Conclusions

The results of the present study indicate that the MIBQ should be considered a reasonable scale to assess maternal emotional involvement with the fetus and infant during pregnancy and in the postpartum period. In addition, the MIBQ has two subscales, LA and AR. The EPDS showed a correlation with both LA and AR. This study suggests associations between depressive symptoms and bonding disorder. These results indicate that the MIBQ is appropriate for pregnant as well as postpartum women to assess maternal bonding toward a fetus and infant.

## Abbreviations

AIC, Akaike Information Criterion; AR, anger and rejection; CFA, confirmatory factor analysis; CFI, comparative fit index; CMIN, chi-squared; DF, degree of freedom; EFA, exploratory factor analysis; EPDS, Edinburgh Postnatal Depression Scale; LA, lack of affection; MAAS, Maternal Antenatal Attachment Scale; MIBQ, Mother-Infant Bonding Questionnaire; PBQ, Postpartum Bonding Questionnaire; RMSEA, root mean square error of approximation; T1, early pregnancy before week 25; T2, late pregnancy around week 36; T3, 5 days after delivery; T4, 1 month after delivery
